# Hepatitis B Vaccination in Senegalese Children: Coverage, Timeliness, and Sociodemographic Determinants of Non-Adherence to Immunisation Schedules (ANRS 12356 AmBASS Survey)

**DOI:** 10.3390/vaccines9050510

**Published:** 2021-05-15

**Authors:** Lauren Périères, Fabienne Marcellin, Gora Lo, Camelia Protopopescu, El Hadji Ba, Marion Coste, Coumba Touré Kane, Gwenaëlle Maradan, Aldiouma Diallo, Cheikh Sokhna, Sylvie Boyer, on behalf of the ANRS 12356 AmBASS Survey Study Group

**Affiliations:** 1 VITROME, Campus IRD-UCAD, CP 18524 Dakar, Senegal; lauren.perieres@etu.univ-amu.fr (L.P.); el-hadj.ba@ird.fr (E.H.B.); aldiouma.diallo@ird.fr (A.D.); 2 Inserm, IRD, SESSTIM, Sciences Economiques & Sociales de la Santé & Traitement de l’Information Médicale, ISSPAM, Aix Marseille University, 13385 Marseille, France; camelia.protopopescu@inserm.fr (C.P.); marion.coste@inserm.fr (M.C.); gwenaelle.maradan@inserm.fr (G.M.); sylvie.boyer@inserm.fr (S.B.); 3 Institut de Recherche en Santé de Surveillance Epidémiologique et de Formation, BP 7325 Diamniadio, Senegal; gora.lo@iressef.org (G.L.); coumba.toure@ucad.edu.sn (C.T.K.); 4 CNRS, EHESS, Centrale Marseille, AMSE, Aix Marseille University, 13001 Marseille, France; 5 ORS PACA, Observatoire Régional de la Santé Provence-Alpes-Côte d’Azur, 13385 Marseille, France; 6 IRD, SSA, VITROME, Aix Marseille University, 13385 Marseille, France; cheikh.sokhna@ird.fr

**Keywords:** birth dose vaccination, hepatitis B vaccine, pentavalent vaccination, Senegal, vaccination timeliness, vaccination coverage

## Abstract

Detailed knowledge about hepatitis B virus (HBV) vaccination coverage and timeliness for sub-Saharan Africa is scarce. We used data from a community-based cross-sectional survey conducted in 2018–2019 in the area of Niakhar, Senegal, to estimate coverage, timeliness, and factors associated with non-adherence to the World Health Organisation-recommended vaccination schedules in children born in 2016 (year of the birth dose (BD) introduction in Senegal) and 2017–2018. Vaccination status was assessed from vaccination cards, surveillance data, and healthcare post vaccination records. Among 241 children with available data, for 2016 and 2017–2018, respectively, 31.0% and 66.8% received the BD within 24 h of birth (BD schedule), and 24.3% and 53.7% received the BD plus at least two pentavalent vaccine doses within the recommended timeframes (three-dose schedule). In logistic regression models, home birth, dry season birth, and birth in 2016 were all associated with non-adherence to the recommended BD and three-dose schedules. Living over three kilometres from the nearest healthcare post, being the firstborn, and living in an agriculturally poorer household were only associated with non-adherence to the three-dose schedule. The substantial proportion of children not vaccinated according to recommended schedules highlights the importance of considering vaccination timeliness when evaluating vaccination programme effectiveness. Outreach vaccination activities and incentives to bring children born at home to healthcare facilities within 24 h of birth, must be strengthened to improve timely HBV vaccination.

## 1. Introduction

Hepatitis B virus (HBV) infection is highly endemic in sub-Saharan Africa, affecting approximately 80 million individuals [1]. It accounts for over 70,000 deaths annually [2]. In 2016, the World Health Assembly adopted the ‘Global health sector strategy on viral hepatitis’, which aims to eliminate viral hepatitis as a public health threat by 2030. The strategy’s targets, which include a 90% reduction in hepatitis B incidence and a 65% reduction in HBV-related mortality by 2030 [3], have been endorsed by most sub-Saharan African countries [4].

HBV transmission in sub-Saharan Africa mainly occurs during early childhood through perinatal transmission and horizontal transmission among children [5]. Horizontal transmission occurs through close contact which is neither perinatal or sexual [5]. Although the mechanisms of horizontal transmission are not fully understood, potential transmission routes include the sharing of personal objects and food [6]. Approximately 90% of HBV infections acquired by infants under six months become chronic [7], with a higher risk of developing cirrhosis and hepatocellular carcinoma [8]. Accordingly, HBV prevention in this sub-population is key to reducing the disease burden in the region.

Vaccination is the cornerstone of HBV prevention. Perinatal infection can be prevented through a monovalent vaccine within 24 h of birth (the so-called ‘birth dose’ or BD hereafter), while additional HBV vaccine doses during early childhood prevent horizontal transmission [9]. Currently, the World Health Organisation (WHO) recommends that all children receive at least three doses of HBV vaccine (the BD within 24 h, followed by two or three additional doses) [9]. Hepatitis B vaccination averted 310 million new HBV cases between 1990 and 2020 [10]. In 2017, although all countries in sub-Saharan Africa had included HBV infant vaccination in their national Expanded Programme on Immunisation (EPI), only nine had introduced the BD, including Senegal [4]. 

HBV infection is a major public health problem in Senegal. In 2016, prevalence in the general population was estimated at 8.1% (95% confidence interval [CI], 7.5–9.0%) [1]. The three-dose pentavalent vaccine (for diphtheria, tetanus, pertussis, HBV, and *Haemophilus influenza type b*, scheduled at 6, 10, and 14 weeks after birth) and the BD monovalent vaccine, were introduced in the country’s national EPI in 2004 and 2016, respectively. As part of the EPI, vaccines are administered for free in public healthcare facilities, as well as during mass immunisation campaigns and door-to-door activities [11,12]. HBV vaccine coverage in Senegal is high according to recent WHO/UNICEF estimates and data in the 2019 Demographic and Health Survey (DHS). An estimated 81% of children born in 2019 received the BD (based on Senegalese government data) [13], and 92% of children born in 2017–2018 received all three doses of the pentavalent vaccine [14]. However, these data are only descriptive. Furthermore, vaccination timeliness was not taken into account in the pentavalent vaccine coverage estimates [14]. Measuring timeliness is essential to evaluate the effectiveness of a vaccination programme, as doses received either too early or too close to one another can result in suboptimal immune protection, [15] while delays in vaccination increase susceptibility to vaccine-preventable diseases [16]. Accordingly, studies on adherence to HBV vaccination schedules which identify barriers to timely vaccination are needed, in order to inform decisions on health policy, with a view of improving vaccination programme effectiveness.

The objectives of the present study were: (i) to estimate the coverage and timeliness of HBV vaccination according to WHO recommendations in a mostly rural area of Senegal, and (ii) to identify individual factors associated with non-adherence to recommended schedules. More specifically, we considered BD within 24 h of birth, and BD within 24 h of birth followed by at least two timely doses of the pentavalent vaccine (as receiving three timely doses provides optimal immune protection against HBV [9,17]).

## 2. Materials and Methods

### 2.1. Study Setting and Design 

The present analysis was conducted as part of the larger ANRS 12356 AmBASS cross-sectional survey, which aimed to assess the health and socioeconomic burden of HBV infection at the individual, household, and population levels, in people living in the area covered by the Niakhar Health and Demographic Surveillance System (HDSS) facility. The HDSS, located 135 kilometres east of Dakar, Senegal, regularly monitors this population, recording births, deaths, migrations, and pregnancies [18]. In 2018, it covered a population of 44,854 individuals in 30 villages [19].

This mostly rural area has four healthcare posts managed by nurses who provide primary care, including maternal and child healthcare, vaccination, and childbirth services [19]. Each post has a specific day every week when the principal activity conducted is vaccination. Vaccination is also performed through regular outreach services for children living in the villages furthest away from the healthcare posts. At each vaccination, the date of the next vaccination appointment is written on the child’s vaccination card and communicated orally to caregivers (i.e., parents, guardians, etc.). Caregivers of children who miss vaccination appointments are contacted by community health volunteers for a new appointment. Children born in healthcare posts are supposed to receive the BD vaccination within 24 h of their birth, in accordance with national and WHO recommendations (BD schedule hereafter) [9,20].

Data collection for AmBASS took place between October 2018 and May 2019. Household sampling was based on a two-stage stratified design, using simple random sampling at both stages. First, the villages of the Niakhar HDSS were divided into 3 semi-urban villages and 27 rural villages, according to their levels of infrastructure. The three semi-urban villages and a random sample of eight rural villages were selected for participation. Second, 401 households in these 11 villages were randomly selected in order to reach the target number of 3200 survey participants. Among the selected households, all individuals residing in the household aged at least six months old were invited to participate. For those under 18 years of age (‘child’ hereafter), a caregiver had to be present in the household at the time of the survey and give consent for the child’s participation. The ANRS 12356 AmBASS survey sampling strategy and methodology is described in detail elsewhere [19].

### 2.2. Data Collection 

Once consent was obtained for a child’s participation, trained fieldworkers collected data on their HBV vaccination status and administered a socioeconomic questionnaire to the caregiver. Data were recorded electronically on tablets using Voxco Survey Software (version 2) [21].

#### 2.2.1. HBV Vaccination Status 

For the present analysis, we used the following three data sources to determine the child’s HBV vaccination status: (i) their vaccination card (if available), (ii) six-monthly vaccination data from the HDSS database, and (iii) vaccination records in healthcare posts (where possible). For children with a vaccination card available at the time of the AmBASS survey, information was compared with HDSS data. Vaccination record information from healthcare posts was used when vaccination dates were different between the vaccination card and the HDSS database, or when the child had no vaccination card available or no vaccination information in the HDSS database.

#### 2.2.2. Socioeconomic Questionnaires 

One socioeconomic questionnaire documented the child’s sociodemographic characteristics, health history (physical and mental impairments, hospitalisations, and illnesses), and exposure to HBV transmission risk factors. Another socioeconomic questionnaire-administered to the child’s caregiver collected information on the sociodemographic characteristics of the latter, as well as on the economic characteristics of their household. 

### 2.3. Ethical Consideration 

The study received ethical approval from the Senegalese National Ethical Committee for Research in Health (no. 082MSAS/DPRS/CNERS), and authorisation from the French Commission on Information Technology and Liberties (reference MMS/HG/OTB/AR181521).

### 2.4. Study Population 

The study population for the present analysis comprised children from the AmBASS survey born on or after 1 January 2016 (i.e., after the introduction of the BD vaccine for hepatitis B in the national EPI).

### 2.5. Study Outcome 

The following binary variables constituted the two study outcomes: (i) non-adherence to the BD within 24 h of birth (BD schedule) and (ii) non-adherence to the BD within 24 h of birth followed by at least two timely doses of the pentavalent vaccine (three-dose schedule hereafter). We considered ‘within 24 h of birth’ as including the day of birth and the next day [22]. For the pentavalent doses, we considered the following WHO-recommended timeframe (which is the same as that recommended in Senegal guidelines): the first pentavalent dose at least six weeks after birth, and at least four weeks between the first and the second pentavalent doses [9,20].

### 2.6. Explanatory Variables 

The following explanatory variables were tested as factors associated with non-adherence to the BD and three-dose schedules, based on previous literature [11,12,22,23,24,25] and available data:

- Children’s sociodemographic characteristics: sex (male/female); season of birth (wet season [July–October]/dry season ([November–June]); place of birth (healthcare facility [i.e., healthcare post, healthcare centre, hospital, etc.]/home); birth order (1/≥2);
- Children’s living conditions: type of village (semi-urban/rural); distance to the nearest healthcare post (≤3 km/>3 km); household living conditions index (1st/2nd/3rd/4th quartile); household agricultural resources index (1st/2nd/3rd/4th quartile or 1st quartile/2nd–4th quartiles). The latter two indexes were built using a multiple correspondence analysis of information on durable goods, agricultural/farming resources, and housing characteristics at the household level;
- Parents’ sociodemographic characteristics: mother’s age at childbirth (≤19/20–29/≥30 years); prenatal consultation during the mother’s most recent pregnancy (yes/no); mother’s marital status (married/not married [single, widowed, divorced]); mother’s educational level (no schooling/primary school/secondary school and higher); father’s educational level (no schooling/primary school/secondary school and higher).

Furthermore, in order to take into account the effect of the progressive implementation of the BD in Senegal, we tested whether being born in the 2016 calendar year (i.e., the year the BD was introduced in the country’s EPI) versus being born in subsequent years (i.e., 2017 or 2018), was associated with non-adherence to the two schedules.

### 2.7. Statistical Analyses

#### 2.7.1. Data Weighting and Calibration 

Data collected in the AmBASS survey were weighted and calibrated to ensure the survey sample was representative of people living in the Niakhar HDSS in terms of sex and age. Sampling weights were calculated as the inverse of the individual probability of inclusion in the sample. Final weights were obtained by multiplying sampling weights by calibration factors, the latter being calculated as the ratio of the percentage of individuals in the HDSS demographic database to the percentage of individuals in the survey sample for each age and sex stratum [19]. Weighted and calibrated data were used for all analyses. 

#### 2.7.2. Descriptive Analyses

We used percentages for categorical variables, and the Chi-square test to compare the characteristics of children who had available vaccination data with those with missing vaccination data.

#### 2.7.3. Regression Models 

We used logistic regression models to identify the factors associated with each one of the two study outcomes. All covariates with a *p*-value below 0.25 (Wald chi-square test) in the univariable analyses were considered eligible for the multivariable analyses. The final multivariable model was constructed using a backward stepwise selection procedure, with a *p*-value threshold set at 0.10. 

We also used a Heckman probit model [26] to test whether differences between children with available vaccination data and those with missing vaccination data could bias the estimations of the two regression analyses.

#### 2.7.4. Sensitivity Analyses

Two sensitivity analyses were conducted to assess the robustness of the results when considering two alternative outcomes, which were defined by changing the criteria for timeliness. For the first outcome, we separately assessed non-adherence to the BD and to the three-dose schedules, using a less restrictive timeliness threshold of seven days after birth (instead of 24 h) for the BD. For the second outcome, we assessed non-adherence to the overall HBV vaccination schedule as set out in Senegal’s EPI. More specifically, the EPI provides for a total of four doses (i.e., the BD and three pentavalent doses), with a minimum of four weeks between the second and third pentavalent dose (WHO and national recommendations [9,20]).

All statistical analyses were performed using Stata software, version 14.2 for Windows (StataCorp, College Station, TX, USA).

## 3. Results

### 3.1. Sociodemographic Characteristics of Children 

A total of 272 children participating in the AmBASS survey were born on or after 1 January 2016. Of these, 88.0% (*n* = 241) had available vaccination data and comprised our study population. Slightly over half (54.5%) were female, 65.5% were born in 2017–2018, 62.0% were born during the dry season, and 79.5% were born in a healthcare facility. The majority (71.0%) lived within three kilometres of the nearest healthcare post. No significant sociodemographic differences were observed between children with available vaccination data and the 31 children with missing vaccination data, except for the year of birth (see Table 1).

### 3.2. Vaccination Coverage and Timeliness

**Figure 1:**
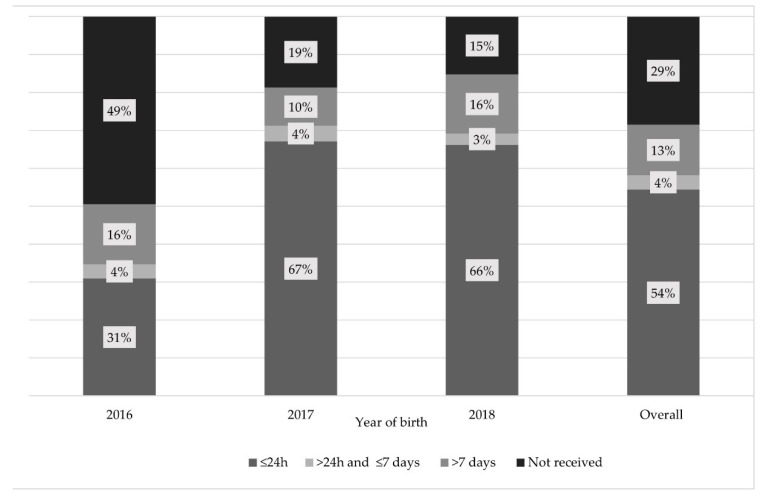
Coverage and administration timeframes of hepatitis B virus (HBV) vaccine birth dose (BD) in children born after its introduction in Senegal’s national Expanded Programme on Immunisation in 2016, per year of birth (percentage of children) (ANRS 12356 AmBASS survey, *n* = 241 children living in the rural area of Niakhar with available vaccination data, using weighted and calibrated data).

Of the 241 children with available vaccination data, 71.5% had received the BD. Of these, 54.5% had received it within 24 h of birth, while 58.2% received it within seven days of birth. Figure 1 shows the BD vaccination administration timeframe according to year of birth. Adherence to the BD schedule within 24 h increased from 31.0% in 2016 (i.e., the year the BD was introduced) to 66.8% in 2017–2018. Furthermore, the proportion of children who received the BD within seven days of birth increased from 34.7% in 2016 to 70.5% in 2017–2018.

**Figure 2:**
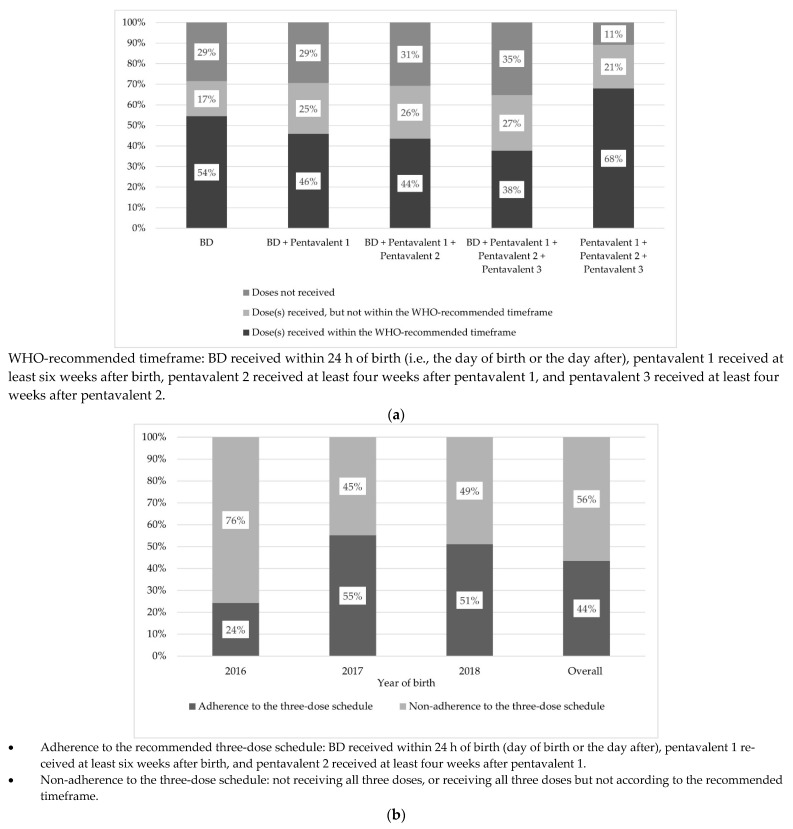
Coverage and timeliness of the WHO-recommended three-dose HBV vaccination schedule (i.e., Birth dose (BD) plus at least two doses of pentavalent vaccine) in children born after the BD was introduced in Senegal’s national Expanded Programme on Immunisation in 2016 (ANRS 12356 AmBASS survey, *n* = 241 children living in the mostly rural area of Niakhar with available vaccination data, using weighted and calibrated data). (**a**) HBV vaccine coverage and timeliness (percentage of children), per dose schedule; (**b**) Adherence and non-adherence (percentage of children) to recommended three-dose HBV vaccine schedule according to year of birth.

Figure 2 shows the level of vaccination timeliness according to all the different dose schedules (Figure 2a) and adherence to the three-dose schedule, per year of birth (Figure 2b). The majority of children who started the vaccine series completed it fully (i.e., BD plus all three pentavalent doses). More specifically, 71.6% of children with available vaccination data received at least three doses of HBV vaccine (BD plus at least two doses of the pentavalent vaccine). However, only 43.6% were vaccinated according to the WHO-recommended schedule (24.3% of those born in 2016 and 53.7% of those born in 2017–2018) mainly because of a >24-h delay between birth and BD administration. Furthermore, although 89.1% of children received all three doses of the pentavalent vaccine, only 68.0% received them according to the national recommended timeframe.

### 3.3. Factors Associated with Non-Adherence to WHO-Recommended Schedules

#### 3.3.1. Non-Adherence to the BD Schedule

Factors associated with non-adherence to the BD schedule in univariable and multivariable logistic regression analyses are presented in Table 2. Variables eligible to enter the multivariable analysis (*p* < 0.25 in univariable analysis) were the child’s sex, season of birth, place of birth, birth order, and the birth year. In multivariable analysis, being born in 2016 (the year the BD was introduced in Senegal’s EPI) (versus 2017–2018) (aOR [adjusted odds ratio] 4.94, 95% CI 2.14–11.40) was associated with non-adherence to the BD schedule at the 5% threshold. Moreover, being born during the dry season (aOR 1.97, 95% CI 0.99–3.95) and at home (versus in a healthcare facility) (aOR 2.02, 95% CI 0.91–4.47) were associated with non-adherence to the BD schedule at the 10% threshold.

#### 3.3.2. Non-Adherence to the Three-Dose Schedule 

Table 3 shows the results of the univariable and multivariable analysis of factors associated with non-adherence to the three-dose schedule. Variables eligible for the multivariable model were the child’s season of birth, place of birth, birth order, distance to the nearest healthcare post, household agricultural wealth, and the birth year. After multivariable adjustment, factors independently associated with non-adherence to the three-dose schedule at the 5% threshold were birth during the dry season (aOR 2.70, 95% CI 1.35–5.39), home birth (versus in a healthcare facility) (aOR 2.38, 95% CI 1.02–5.56), living in an agriculturally poorer household (1st wealth quartile versus 2nd–4th wealth quartiles) (aOR 3.18, 95% CI 1.33–7.61), and being born in 2016 (versus 2017–2018) (aOR 3.93, 95% CI 1.74–8.89). Living more than three kilometres away from the nearest healthcare post (aOR 2.04, 95% CI 0.97–4.27) and being the first-born child (aOR 2.07, 95% CI 0.97–4.27) were both associated with non-adherence at the 10% threshold.

The Heckman model did not have sufficient power to estimate the correction for selection bias in the analysis (data not shown). The only factor significantly associated with unavailable vaccination data was year of birth (Table 1). 

#### 3.3.3. Sensitivity Analyses

Using a less restrictive timeframe for BD administration (within seven days of birth instead of 24 h), the factors associated with non-adherence to both the BD (Table S1) and three-dose schedules (Table S2) were similar to those in the Section 3.3.1 and Section 3.3.2. For the three-dose schedule only, being the first-born child was not associated in multivariable analysis (Table S2). When considering the overall four-dose HBV vaccination schedule (i.e., the BD and all three pentavalent doses, as set out in Senegal’s EPI) as the outcome variable, the results of the multivariate model were also similar, except that neither being born during the dry season nor being the first-born child were independently associated with non-adherence (Table S3).

## 4. Discussion

By measuring the timeliness of HBV vaccination in Senegal, this study is one of the first to assess not only the coverage but also the effectiveness of a HBV vaccination programme in sub-Saharan Africa [27]. It is also the first study in Senegal, and one of the few in sub-Saharan Africa to date, to identify barriers to both BD timeliness and complete HBV vaccination. Adherence to the WHO’s recommended HBV vaccination schedules significantly increased between 2016 and 2017–2018 (from 31.0% to 66.8% for the BD schedule, and from 24.3% to 53.7% for the three-dose schedule). Being born at home, during the dry season, and in 2016 (i.e., the year the BD was introduced in Senegal’s EPI) were three distinct factors independently associated with non-adherence to both the BD schedule and three-dose schedules. Additional correlates of non-adherence to the three-dose schedule included living more than three kilometres away from the nearest healthcare post, being the first-born child, and living in an agriculturally poorer household. 

The proportion of children who received the BD within 24 h of birth (i.e., as per the WHO recommendation) in our study was slightly lower than estimates in the 2018 and 2019 Senegalese DHS, which estimated that 54% of children born between mid-2016 and December 2017 [28], and 81.3% of those born between mid-2017 and December 2018 [14] received the BD within 24 h of birth. Although our results are not nationally representative, our estimates for 2016 are lower than WHO/UNICEF estimates (31% versus 58%), but comparable for 2017 (67% versus 72%) and 2018 (63% versus 66%) [13]. BD coverage and timeliness in our study were significantly lower in 2016 than in 2017 and 2018, most likely because of the progressive vaccine roll-out in 2016. Moreover, BD coverage and timeliness were slightly lower in 2018 than in 2017, most probably due to the healthcare workers’ strike from April to December 2018, which affected vaccination services throughout the country. Compared with other countries in the region, the proportion of children with timely BD vaccination is high in Senegal. According to a recent systematic review, only 1.3% (95% CI, 0.0–4.5%) and 21.5% (95% CI, 9.4–36.8%) of children in sub-Saharan African countries that have introduced the BD received it within 24 h and seven days of birth, respectively [27]. 

To our knowledge, no other study to date has estimated the coverage or timeliness of the WHO-recommended HBV vaccination schedule in children in sub-Saharan Africa. The large disparity we found between the proportion of children who received all three doses (71.6%), and the proportion who received them according to the recommended timeframe (43.6%) is mainly explained by the >24 h delay between birth and BD administration. This disparity reflects findings in studies evaluating infant HBV three-dose pentavalent vaccination coverage and timeliness in Kenya [29,30], Burkina Faso [24], Ghana [31], Cameroon [25], and Ethiopia [32]. For instance, in a study in Burkina Faso, while 93% of children born between 2006–2008 received the third pentavalent vaccine dose, only 48% received it on time (in that study, timely vaccination was considered as receiving the third dose anytime from 3 days prior to the recommended vaccination date to 28 days after it) [24]. Moreover, a previous study, based on data from the 2014 Senegal DHS, found that only 55% of children in the country received the third dose of pentavalent vaccine on time [33].

We tested a wide range of individual sociodemographic and economic factors for their association with non-adherence to the recommended BD and three-dose HBV vaccination schedules. Most of the risk factors associated with non-adherence were also identified in previous studies. Moreover, results from several studies reflect our finding of the association between the child’s place of birth and HBV vaccine receipt and timeliness. In Kenya, children born in healthcare facilities were 1.25 (95% CI 1.22–1.28) times more likely to receive all three pentavalent doses according to recommended age-appropriate timeframes than those born at home [30]. In Cameroon, children born in healthcare facilities were 2.11 (95% CI 1.69–2.64) times more likely to receive all three pentavalent doses (irrespective of vaccination timeliness) than those born at home [25]. These two findings may be due—at least in part—to the possibility that women who give birth in healthcare facilities in these two countries are more frequent users of these services, and therefore, are more likely to be aware of the vaccination schedule [30]. However, unlike our study, the child’s place of birth was not associated with adherence to the BD schedule in a study focusing on The Gambia [22] or in a systematic review of the coverage and timeliness of BD vaccination in sub-Saharan Africa [27]. In Senegal, the monovalent HBV BD vaccine is free of charge and widely available in all healthcare facilities providing childbirth, maternal, and child health services. Furthermore, mothers who give birth at home are encouraged to go to the healthcare posts as soon as possible afterwards so their child can receive the BD vaccine. However, in another study conducted in the area covered by the Niakhar HDSS (i.e., the same area as our present study), healthcare workers mentioned that home births were a barrier to timely vaccination at birth, as mothers may encounter various difficulties to bring their child to a health facility within 24 h of birth [34]. 

We found that living more than 3 km from a healthcare post was associated with non-adherence to the three-dose schedule. This result echoes findings in a Kenyan study where distance to a healthcare facility was associated with age-appropriate receipt of the third dose of pentavalent vaccine [30]. In a study in rural Ethiopia, time—which might be considered as a proxy for distance to healthcare facilities—was associated with three-dose pentavalent receipt [35]. Geographical distance from healthcare facilities may be a barrier for childhood vaccination—particularly the administration of the BD within 24 h of birth—in remote areas [35], for many reasons, including caregiver availability and cost of transport. 

Being the first-born child was associated with non-adherence to the hepatitis B vaccination in our study. Similarly, being the third-born child and above (versus first-or second-born) was associated with up-to-date and age-appropriate receipt of three doses of pentavalent vaccine in Kenya [30]. Children with an older sibling may be better vaccinated as multiparous mothers may already be more aware of vaccination schedules through their experience with their first child. 

We also identified risk factors associated with non-adherence to HBV vaccination that have not been previously reported in the literature. For instance, we found that the season of birth (dry/wet) was associated with non-adherence to the BD schedule, which contrasts with the results of the above-mentioned study in The Gambia [22]. Seasonal migration during the dry season, particularly of women, may partly explain lower HBV vaccination coverage and timeliness in the Niakhar HDSS area. 

Living in an agriculturally poorer household was associated with poorer adherence to the recommended three-dose schedule in our study. However, we did not identify any other study exploring this association in sub-Saharan Africa. This result suggests that although EPI vaccines are free in Senegal, the poorest agricultural households may face additional barriers to vaccinating their children on time.

Finally, being born in 2016 (i.e., the year the BD vaccine was introduced in Senegal’s EPI) (versus being born in 2017–2018) was associated with non-adherence to both the recommended BD and three-dose schedules. This reflects a positive trend in the vaccination programme performance in the study area in recent years. 

The ‘Global health sector strategy on viral hepatitis’ aims to achieve a 90% infant vaccination coverage rate, and a BD vaccination rate of 80% coverage by 2030 [3]. Our results highlight the importance of complementing coverage estimations with measurements of timeliness [30,31,32,33] in order to assess the effectiveness of vaccination programmes more accurately. Traditionally, effectiveness has been assessed by measuring the proportion of children who receive a vaccine by a benchmark age, irrespective of the timing of the vaccination. However, overall high vaccination coverage can mask serious delays in vaccine administration, which may in turn undermine a programme’s effectiveness [36]. 

Our present study identified several at-risk populations (children born at home, those living more than three kilometres from a healthcare centre, those born during the dry season, first-born children, and those born in an agriculturally poorer household) that need to be targeted in order to improve vaccination timeliness. Encouraging and increasing childbirth in healthcare facilities could be a very effective strategy to improve both HBV vaccination timeliness and coverage. In addition, when mothers give birth at home, positive incentives are required to further encourage them to bring their child to a healthcare structure for HBV vaccination within 24 h of birth. Coverage and timeliness of doses after the BD dose may also be improved through outreach immunisation activities, especially for children who live furthest away from healthcare facilities [27]. Moreover, improved communication models are needed to both increase mothers’ awareness of the importance of respecting the vaccination schedule in order to prevent HBV transmission to their new-borns, and to help them to remember their vaccination appointments. A recent cluster-randomised controlled trial in rural Kenya found that mobile phone-delivered reminders with a small monetary incentive significantly improved coverage and timeliness of routine vaccines [37]. Redesigned immunisation reminder cards may also help to increase vaccination coverage and timeliness [38]. 

This study has several limitations. First, we may have slightly overestimated vaccination coverage as we expected that children without available vaccination data had poorer vaccination status and different socioeconomic characteristics than those of children with available data. However, the only factor significantly associated with missing vaccination data was birth year. Accordingly, we can assume that the missing data were almost completely randomly distributed, and therefore, the selection bias was probably negligible in our regression analyses.

Second, although we explored a large range of individual factors, we did not investigate determinants related to the healthcare services, such as logistic issues related to vaccines, healthcare worker knowledge, or issues related to information delivery [39,40], all of which may play a role in non-adherence to HBV vaccination schedules. Further research is needed to better understand and disentangle the specific role of individual and healthcare-related supply factors in vaccination programme effectiveness.

Third, although we used the WHO-recommended HBV vaccine timeframe as the schedule of reference (which is the same as that recommended in Senegal guidelines), there is no universally accepted standard for timeliness and official vaccination schedules may differ from one country to another. Accordingly, inter-study comparison is difficult [36]. However, we conducted sensitivity analyses to investigate the effects of using both more and less restrictive criteria to define timeliness. Results found that overall, the findings were similar to those for the main analysis. 

## 5. Conclusions

Although our data show the progress made in recent years in HBV vaccine coverage for children in rural Senegal, a substantial proportion of children had not been vaccinated according to the WHO-recommended timeframe. Incentives to bring all children born at home to healthcare facilities within 24 h of birth could be very effective to increase timely BD coverage. Outreach vaccination activities must also be strengthened to increase coverage in children born at home, those living far from healthcare posts, and those in agriculturally poorer households. In addition, vaccination timeliness should be considered when evaluating the effectiveness of the current HBV vaccination programme in Senegal.

## Figures and Tables

**Table 1:** Characteristics of children born after 2016 participating in the ANRS 12356 AmBASS survey and comparison between those with available vaccination data (*n* = 241) and those with missing vaccination data (*n* = 31), using weighted and calibrated data.

Characteristics (% of Missing Data)	Overall (*n* = 272) %	Available Vaccination Data (*n* = 241) %	Missing Vaccination Data (*n* = 31) %	Pearson Chi2 *p* Value
**Sex** (0.0)				
Male	47.3	45.5	61.1	0.122
Female	52.7	54.5	38.9	
**Season of birth** (0.0)				
Wet season	35.8	38.0	19.7	0.058
Dry season	64.2	62.0	80.3	
**Place of birth** (0.0)				
Healthcare facility	77.6	79.5	63.2	0.155
Home	22.4	20.5	36.8	
**Birth order** (0.6)				
1	19.9	18.5	30.5	0.132
≥2	80.1	81.5	69.5	
**Type of village** (0.0)				
Semi-urban	57.7	58.7	50.0	0.475
Rural	42.3	41.3	50.0	
**Distance to closest healthcare post** (0.6)				
≤3 km	69.6	71.0	60.0	0.268
>3 km	30.4	29.0	40.0	
**Mother’s age at child’s birth (years)** (5.7)				
≤19	6.9	6.1	15.3	0.119
20–29	46.0	48.1	22.9	
≥30	47.1	45.8	61.8	
**Prenatal consultation during the mother’s most recent pregnancy** (14.2)				
Yes	96.2	95.9	100.0	0.444
No	3.8	4.1	0.0	
**Mother’s marital status** (11.2)				
Married	97.1	96.9	100.0	0.439
Not married (single, widowed, divorced)	2.9	3.1	0.0	
**Mother’s educational level** (6.9)				
No schooling	66.5	68.0	49.6	
Primary school	20.1	18.7	35.6	0.394
Secondary school and higher	13.4	13.3	14.8	
**Father’s educational level** (25.4)				
No schooling	57.3	57.9	50.8	
Primary school	26.6	24.9	44.5	0.263
Secondary school and higher	16.1	17.2	4.7	
**Household living conditions index^1^** (0.0)				
1st quartile	13.7	13.1	18.0	
2nd quartile	22.6	21.6	29.6	0.354
3rd quartile	26.0	27.7	13.2	
4th quartile	37.7	37.6	39.2	
**Household agricultural wealth index^1^** (0.0)				
1st quartile	24.1	21.4	44.2	0.118
2nd quartile	18.7	19.1	15.8	
3rd quartile	24.3	25.6	14.3	
4th quartile	32.9	33.9	25.7	
**Born in 2016** (0.0)				
Yes	39.3	34.5	74.1	0.006
No (2017–2018)	60.7	65.5	25.9	

^1^ The household living conditions index and the agricultural wealth index were built using a multiple correspondence analysis of information on durable goods, agricultural and farming resources, and housing characteristics at the household level.

**Table 2:** Factors associated with non-adherence to the hepatitis B virus (HBV) birth dose (BD) schedule in children born on or after 1 January 2016 living in the mostly rural area of Niakhar (*n* = 241 children participating in the ANRS 12356 AmBASS survey with available vaccination data, logistic regression models using weighted and calibrated data).

Characteristics (% of Missing Data)	Non-Adherence to the BD Schedule (*n* = 112) %	Adherence to the BD Schedule (*n* = 129) %	Univariable Analysis		Multivariable Analysis	
			OR [95% CI]	*p* Value	aOR [95% CI]	*p* Value
**Sex** (0.0)						
Male (ref.)	40.1	49.9	1	0.200		
Female	59.9	50.1	1.49 [0.78–2.84]			
**Season of birth** (0.0)						
Wet season (ref.)	31.3	43.6	1	0.102	1	0.054
Dry season	68.7	56.4	1.70 [0.88–3.27]		1.97 [0.99–3.95]	
**Place of birth** (0.0)						
Healthcare facility (ref.)	74.4	83.8	1	0.108	1	0.077
Home	25.6	16.2	1.78 [0.86–3.72]		2.02 [0.91–4.47]	
**Birth order** (0.7)						
1	23.4	14.4	1.81 [0.81–4.06]			
≥2 (ref.)	76.6	85.6	1	0.131		
**Type of village** (0.0)						
Semi-urban (ref.)	58.3	59.0	1	0.922		
Rural	41.7	41.0	1.03 [0.53–2.01]			
**Distance to closest healthcare post** (0.7)						
≤3 km (ref.)	68.9	72.7	1	0.554		
>3 km	31.1	27.3	1.20 [0.61–2.38]			
**Mother’s age at child’s birth (years)** (1.5)						
≤19	8.4	4.1	2.47 [0.48–12.73]			
20–29	50.4	46.1	1.32 [0.70–2.49]	0.461		
≥30 (ref.)	41.2	49.8	1			
**Prenatal consultation during the mother’s most recent pregnancy** (9.9)						
Yes (ref.)	95.4	96.3	1	0.801		
No	4.6	3.7	1.25 [0.18–8.40]			
**Mother’s marital status** (11.2)						
Married (ref.)	95.5	98.0	1	0.341		
Not married (single, widowed, divorced)	4.5	2.0	2.34 [0.35–15.82]			
**Mother’s educational level** (2.9)						
No schooling (ref.)	62.7	72.3	1			
Primary school	23.2	15.0	1.78 [0.76–4.17]	0.363		
Secondary school and higher	14.1	12.7	1.28 [0.54–3.06]			
**Father’s educational level** (22.6)						
No schooling (ref.)	59.7	56.4	1			
Primary school	27.2	23.0	1.12 [0.56–2.32]	0.598		
Secondary school and higher	13.1	20.6	0.60 [0.16–2.30]			
**Household living conditions index^1^** (0.0)						
1st quartile	16.8	10.0	2.20 [0.90–5.38]	0.424		
2nd quartile	21.7	21.5	1.31 [0.48–3.63]			
3rd quartile	29.2	26.5	1.44 [0.67–3.09]			
4th quartile (ref.)	32.3	42.0	1			
**Household agricultural wealth index^1^** (0.0)						
1st quartile	25.0	18.4	1.63 [0.64–4.17]	0.558		
2nd quartile	21.3	17.2	1.48 [0.60–3.65]			
3rd quartile	23.1	27.7	1.00 [0.47–2.14]			
4th quartile (ref.)	30.6	36.7	1			
**Born in 2016** (0.0)						
Yes (ref.)	52.3	19.6	4.48 [1.99–10.08]	0.002	4.94 [2.14–11.40]	0.002
No (2017–2018)	47.7	80.4	1		1	

OR = odds ratio, aOR = adjusted odds ratio, CI = confidence interval, BD = birth dose. ^1^ The household living conditions index and the agricultural wealth index were built using a multiple correspondence analysis of information on durable goods, agricultural and farming resources, and housing characteristics at the household level.

**Table 3:** Factors associated with non-adherence to the WHO-recommended three-dose hepatitis B virus (HBV) vaccine schedule in children born on or after 1 January 2016, living in the mostly rural area of Niakhar (*n* = 241 children participating in the ANRS 12356 AmBASS survey with available vaccination data, logistic regression models using weighted and calibrated data).

Characteristics (% of Missing Data)	Non-Adherence to the Three-Dose Schedule (*n* = 138)	Adherence to the Three-Dose Schedule (*n* = 103)	Univariable Analysis		Multivariable Analysis	
			OR [95% CI]	*p* Value	aOR [95% CI]	*p* Value
**Sex** (0.0)						
Male (ref.)	43.6	47.8	1	0.596		
Female	56.4	52.2	1.19 [0.59–2.38]			
**Season of birth** (0.0)						
Wet season (ref.)	30.5	47.7	1	0.034	1	0.010
Dry season	69.5	52.3	2.08 [1.07–4.02]		2.70 [1.35–5.39]	
**Place of birth** (0.0)						
Healthcare facility (ref.)	75.3	85.0	1	0.114	1	0.047
Home	24.7	15.0	1.86 [0.83–4.16]		2.38 [1.02–5.56]	
**Birth order** (0.7)						
1	23.6	12.0	2.27 [0.98–5.24]	0.054	2.07 [0.97–4.40]	0.057
≥2 (ref.)	76.4	88.0	1		1	
**Type of village** (0.0)						
Semi-urban (ref.)	60.4	56.6	1	0.608		
Rural	39.6	43.4	0.86 [0.44–1.66]			
**Distance to closest healthcare post** (0.7)						
≤3 km (ref.)	67.3	75.8	1	0.030	1	0.058
>3 km	32.7	24.2	1.52 [0.81–2.85]		2.04 [0.97–4.27]	
**Mother’s age at child’s birth (years)** (1.5)						
≤19	8.6	2.9	3.64 [0.62–21.3]			
20–29	49.6	46.1	1.32 [0.70–2.47]	0.321		
≥30 (ref.)	41.8	51.0	1			
**Prenatal consultation during the mother’s most recent pregnancy** (8.3)						
Yes (ref.)	96.3	95.4	1	0.778		
No	3.7	4.6	0.78 [0.12–5.13]			
**Mother’s marital status** (11.2)						
Married (ref.)	96.3	97.5	1	0.650		
Not married (single, widowed, divorced)	3.7	2.5	1.49 [0.22–10.01]			
**Mother’s educational level** (2.9)						
No schooling (ref.)	66.9	69.5	1			
Primary school	20.0	17.0	1.23 [0.49–3.06]	0.894		
Secondary school and higher	13.1	13.5	1.01 [0.43–2.27]			
**Father’s educational level** (22.6)						
No schooling (ref.)	62.1	52.4	1			
Primary school	25.1	27.4	0.85 [0.39–1.88]	0.541		
Secondary school and higher	12.8	22.9	0.47 [0.12–1.90]			
**Household living conditions index^1^** (0.0)						
1st quartile	17.4	7.6	2.50 [0.99–6.32]	0.324		
2nd quartile	19.2	24.7	0.85 [0.31–2.36]			
3rd quartile	27.3	28.3	1.05 [0.53–2.08]			
4th quartile (ref.)	36.1	39.4	1			
**Household agricultural wealth index^1^** (0.0)						
1st quartile	26.3	15.1	2.00 [0.95–4.22]	0.064	3.18 [1.33–7.61]	0.015
2nd–4th quartile (ref.)	73.7	84.9	1		1	
**Born in 2016** (0.0)						
Yes (ref.)	46.3	19.2	3.61 [1.68–7.75]	0.004	3.93 [1.74–8.89]	0.004
No (2017–2018)	53.7	80.8	1		1	

OR = odds ratio, aOR = adjusted odds ratio, CI = confidence interval. ^1^ The household living conditions index and the agricultural wealth index were built using a multiple correspondence analysis of information on durable goods, agricultural and farming resources, and housing characteristics at the household level.

## Data Availability

Data available on request due to privacy/ethical reasons.

## Supplementary Materials

The following are available online at https://www.mdpi.com/article/10.3390/vaccines9050510/s1, Table S1: Factors associated with non-adherence to the hepatitis B virus (HBV) birth dose administration within 7 days of birth in children born on or after 1 January 2016 living in the mostly rural area of Niakhar (*n* = 241 children participating in the ANRS 12356 AmBASS survey with available vaccination data, logistic regression models using weighted and calibrated data), Table S2: Factors associated with non-adherence to the three-dose hepatitis B virus (HBV) vaccine schedule (birth dose and two-dose pentavalent, with the birth dose received >7 days/not received) in children born on or after 1 January 2016 living in the mostly rural area of Niakhar (*n* = 241 children participating in the ANRS 12356 AmBASS survey with available vaccination data, logistic regression models using weighted and calibrated data), Table S3: Factors associated with non-adherence to the four-dose hepatitis B virus (HBV) vaccine schedule in children born on or after 1 January 2016 living in the mostly area of Niakhar (*n* = 241 children participating in the ANRS 12356 AmBASS survey with available vaccination data, logistic regression models using weighted and calibrated data).
