# Supplementary material for: Hepatitis B Vaccination in Senegalese Children: Coverage, Timeliness, and Sociodemographic Determinants of Non-Adherence to Immunisation Schedules (ANRS 12356 AmBASS Survey)
Source: Vaccines (Basel). 2021 May 15;9(5):510. doi: 10.3390/vaccines9050510 (PMC8155976; doi:10.3390/vaccines9050510)
Supplement: Supplementary file 1 [file vaccines-09-00510-s001.zip › vaccines-1189071-supplementary.pdf]

Table S1. Factors associated with non-adherence to the hepatitis B virus (HBV) birth dose administration within 7 days of birth in children born on or after 1 January 2016 living in the mostly rural area of Niakhar (*n* = 241 children participating in the ANRS 12356 AmBASS survey with available vaccination data, logistic regression models using weighted and calibrated data).

**Characteristics (% of missing data)**	**BD received >7 days after birth/not received (*n* =101) %**	**BD received ≤ 7 days after birth (*n* =140) %**	**Univariable analysis**		**Multivariable analysis**	
			**OR [95% CI]**	***P* value**	**aOR [95% CI]**	***P* value**
**Sex** (0.0)						
Male (ref.)	39.1	50.0	1	0.157		
Female	60.9	50.0	1.56 [0.81–1.98]			
**Season of birth** (0.0)						
Wet season (ref.)	31.8	42.5	1	0.142	1	0.077
Dry season	68.2	57.5	1.59 [0.83–3.05]		1.84 [0.92–3.67]	
**Place of birth** (0.0)						
Healthcare facility (ref.)	72.9	84.3	1	0.067	1	0.045
Home	27.1	15.7	1.99 [0.94–4.20]		2.29 [1.02–5.13]	
**Birth order** (0.7)						
1	21.2	16.5	1.36 [0.61–3.05]			
≥2 (ref.)	78.8	83.5	1	0.407		
**Type of village** (0.0)						
Semi-urban (ref.)	56.2	60.5	1	0.580		
Rural	43.8	39.5	1.19 [0.59–2.40]			
**Distance to closest healthcare post** (0.7)						
≤3 km (ref.)	68.7	72.6	1	0.574		
>3 km	31.3	27.4	1.20 [0.58–2.53]			
**Mother's age at child's birth (years)** (1.5)						
≤19	7.5	5.1	1.66 [0.35–7.78]			
20–29	49.9	46.7	1.21 [0.65–2.46]	0.729		
≥30 (ref.)	42.6	48.2	1			
**Prenatal consultation during mother's most recent pregnancy** (9.9)						
Yes (ref.)	98.0	94.6	1	0.259		
No	2.0	5.4	0.36 [0.05-2.47]			

**Mother's marital status** (11.2)						
Married (ref.)	95.0	98.1	1	0.264		
Not married (single, widowed, divorced)	5.0	1.9	2.72 [0.41-18.18]			
**Mother's educational level** (2.9)						
No schooling (ref.)	65.7	69.7	1			
Primary school	22.7	15.9	1.52 [0.65–3.58]	0.529		
Secondary school and higher	11.6	14.4	0.85 [0.38–1.94]			
**Father's educational level** (22.6)						
No schooling (ref.)	62.1	54.7	1			
Primary school	25.0	24.9	0.88 [0.42–1.85]	0.664		
Secondary school and higher	12.9	20.4	0.56 [0.14–2.15]			
**Household living conditions index** [1] (0.0)						
1st quartile	17.5	9.95	2.37 [0.90–6.28]			
2nd quartile	22.9	20.7	1.49 [0.53–4.21]	0.423		
3rd quartile	28.4	27.2	1.41 [0.61–3.27]			
4th quartile (ref.)	31.2	42.1	1			
**Household agricultural wealth index** [1] (0.0)						
1st quartile	24.0	19.5	1.53 [0.55–4.25]			
2nd quartile	21.7	17.3	1.56 [0.60–4.03]	0.745		
3rd quartile	24.5	26.4	1.15 [0.51–2.57]			
4th quartile (ref.)	29.8	36.8	1			
**Born in 2016** (0.0)						
Yes (ref.)	53.8	20.6	4.50 [2.05–9.86]	0.002	4.97 [2.21–11.20]	0.002
No (2017-2018)	46.2	79.4	1		1	

OR = odds ratio; Aor = adjusted odds ratio; CI = confidence interval; [1] The household living conditions index and the agricultural wealth index were built using a multiple correspondence analysis of information on durable goods, agricultural and farming resources, and housing characteristics at the household level.

Table S2. Factors associated with non-adherence to the three-dose hepatitis B virus (HBV) vaccine schedule (birth dose and two-dose pentavalent, with the birth dose received >7 days/not received) in children born on or after 1 January 2016 living in the mostly rural area of Niakhar (*n* = 241 children participating in the ANRS 12356 AmBASS survey with available vaccination data, logistic regression models using weighted and calibrated data).

Characteristics (% of missing data)	Non-adherence to the three-dose schedule (*n* =129)	Adherence to the three-dose schedule (*n* = 112)	Univariable analysis		Multivariable analysis	
			OR [95% CI]	P value	aOR [95% CI]	P value
**Sex** (0.0)						
Male (ref.)	42.3	48.9	1	0.424		
Female	57.5	51.1	1.29 [0.65–2.59]			
**Season of birth** (0.0)						
Wet season (ref.)	31.1	46.0	1	0.054	1	0.014
Dry season	68.9	54.0	1.88 [0.99-3.58]		2.43 [1.26-4.69]	
**Place of birth** (0.0)						
Healthcare facility (ref.)	74.5	85.2	1	0.077	1	0.045
Home	25.5	14.8	1.98 [0.91-4.28]		2.32 [1.03–5.23]	
**Birth order** (0.7)						
1	21.6	14.9	1.57 [0.69–3.53]			
≥2 (ref.)	78.4	85.1	1	0.244		
**Type of village** (0.0)						
Semi-urban (ref.)	59.4	57.9	1	0.847		
Rural	40.6	42.1	0.94 [0.48–1.85]			
**Distance to closest healthcare post** (0.7)						
≤3 km (ref.)	67.4	75.0	1	0.236	1	0.074
>3 km	32.6	25.0	1.45 [0.75–2.82]		2.02 [0.92–4.43]	
**Mother's age at child's birth (years)** (1.5)						
≤19	8.6	2.9	3.64 [0.62-21.26]			
20–29	49.6	46.1	1.32 [0.70-2.47]	0.321		
≥30 (ref.)	41.8	51.0	1			
**Prenatal consultation during mother's most recent pregnancy** (9.9)						
Yes (ref.)	98.4	93.3	1	0.108		
No	1.6	6.7	0.22 [0.03-1.49]			
**Mother's marital status** (11.2)						
Married (ref.)	96.1	97.7	1	0.543		
Not married (single, widowed, divorced)	3.9	2.3	1.70 [0.26-11.30]			
**Mother's educational level** (2.9)						
No schooling (ref.)	68.5	67.5	1			
Primary school	19.9	17.3	1.13 [0.46-2.82]	0.731		

Secondary school and above	11.6	15.2	0.76 [0.33-1.72]			
**Father's educational level** (22.6)						
No schooling (ref.)	63.1	51.5	1			
Primary school	23.6	26.5	0.73 [0.33–1.60]	0.532		
Secondary school and above	13.3	22.0	0.35 [0.12–2.00]			
**Household living conditions index**[1] (0.0)						
1st quartile	17.7	7.8	2.51 [1.03-6.13]			
2nd quartile	19.7	23.8	0.92 [0.33-2.55]	0.292		
3rd quartile	26.8	28.7	1.04 [0.50-2.15]			
4th quartile (ref.)	35.7	39.7	1			
**Household agricultural wealth index**[1] (0.0)						
1st quartile	26.6	15.5	1.97 [0.94-4.15]	0.069	3.22 [1.29–8.02]	0.013
2nd–4th quartile (ref.)	73.4	84.5	1		1	
**Year of birth** (0.0)						
2016	46.5	20.8	3.32 [1.60-6.86]	0.005	3.74 [1.70-8.22]	0.004
2017–2018 (ref.)	53.5	79.2	1		1	

OR = odds ratio; aOR= adjusted odds ratio; CI= confidence interval; [1] The household living conditions index and the agricultural wealth index were built using a multiple correspondence analysis of information on durable goods, agricultural and farming resources, and housing characteristics at the household level.

Table S3. Factors associated with non-adherence to the four-dose hepatitis B virus (HBV) vaccine schedule in children born on or after 1 January 2016 living in the mostly area of Niakhar (*n* = 241 children participating in the ANRS 12356 AmBASS survey with available vaccination data, logistic regression models using weighted and calibrated data)

**Characteristics** (% of missing data)	**Non-adherence to the four-dose schedule (*n* = 151)**	**Adherence to the four-dose schedule (*n* = 90)**	**Univariable analysis**		**Multivariable analysis**	
			**OR [95% CI]**	***P* value**	**aOR [95% CI]**	***P* value**
**Sex** (0.0)						
Male (ref.)	45.3	45.8	1	0.946		
Female	54.7	54.2	1.02 [0.49–2.16]			
**Season of birth** (0.0)						
Wet season (ref.)	34.4	44.0	1	0.274		
Dry season	65.6	56.0	1.49 [0.69–3.26]			
**Place of birth** (0.0)						
Healthcare facility (ref.)	74.6	87.6	1	0.037	1	0.028
Home	25.4	12.4	2.41 [1.07–5.42]		2.70 [1.15–6.35]	
**Birth order** (0.67)						
1	21.3	13.8	1.68 [0.71–3.99]			
≥2 (ref.)	78.7	86.2	1	0.205		

**Type of village** (0.0)						
Semi-urban (ref.)	61.2	54.6	1	0.402		
Rural	38.8	45.4	0.76 [0.38–1.53]			
**Distance to closest healthcare post** (0.7)						
≤3 km (ref.)	66.9	77.7	1	0.083	1	0.049
>3 km	33.1	22.3	1.73 [0.92–3.25]		2.12 [1.00–4.50]	
**Mother's age at child's birth (years)** (1.52)						
≤19	7.8	3.4	2.87 [0.47–17.70]			
20–29	50.1	44.6	1.39 [0.70–2.76]	0.444		
≥30 (ref.)	42.1	52.0	1			
**Prenatal consultation during mother's most recent pregnancy** (8.3)						
Yes (ref.)	96.8	94.7	1	0.534		
No	3.2	5.3	0.59 [0.09-3.73]			
**Mother's marital status** (11.2)						
Married (ref.)	95.5	99.0	1	0.172		
Not married (single, widowed, divorced)	4.5	1.0	4.73 [0.44-50.41]			
**Mother's educational level** (2.9)						
No schooling (ref.)	65.6	71.9	1			
Primary school	21.4	14.3	1.63 [0.69–3.88]	0.515		
Secondary school and higher	13.0	13.8	1.03 [0.43–2.45]			
**Father's educational level** (22.6)						
No schooling (ref.)	62.0	51.2	1			
Primary school	25.0	24.9	0.83 [0.34–2.01]	0.521		
Secondary school and higher	13.0	23.9	0.45 [0.11–1.88]			
**Household living conditions index[1]** (0.0)						
1st quartile	16.8	6.9	2.36 [0.95–5.86]			
2nd quartile	19.1	25.9	0.72 [0.25–2.07]	0.286		
3rd quartile	26.1	30.3	0.84 [0.43–1.64]			
4th quartile (ref.)	38.0	36.9	1			
**Household agricultural wealth index[1]** (0.0)						
1st quartile	26.0	13.8	2.18 [1.05–4.56]	0.039	3.59 [1.58–8.18]	0.007

2nd–4th quartile (ref.)	74.0	86.2	1		1	
**Year of birth** (0.0)						
2016	43.0	20.5	2.93 [1.31–6.54]	0.014	3.09 [1.29–7.39]	0.017
2017–2018 (ref.)	57.0	79.5	1		1	

OR = odds ratio; aOR= adjusted odds ratio ; CI= confidence interval; [1] The household living conditions index and the agricultural wealth index were built using a multiple correspondence analysis of information on durable goods, agricultural and farming resources, and housing characteristics at the household level.
